# Kimura’s disease with recurrent bilateral lacrimal gland involvement in a male Japanese child successfully treated with cyclosporine A

**DOI:** 10.1186/s13223-021-00549-y

**Published:** 2021-05-17

**Authors:** Keisuke Sugimoto, Takuji Enya, Yuichi Morimoto, Rina Oshima, Kohei Miyazaki, Mitsuru Okada

**Affiliations:** Department of Pediatrics, Kindai University Faculty of Medicine, 377-2 Ohno-higashi, Osakasayama, Osaka 589-8511 Japan

**Keywords:** Kimura’s disease, Cyclosporine, Pediatrics, Lacrimal gland

## Abstract

**Background:**

Kimura’s disease (KD) is a rare chronic inflammatory disease of unknown etiology. Clinically, KD is characterized by nodular subcutaneous masses, that are typically localized to the neck and head. Involvement of the lacrimal glands and limbs is uncommon and seldom reported.

**Case presentation:**

We report a case of a 4-year-old Japanese boy presenting with bilateral upper eyelid swelling with nodular subcutaneous lesions and peripheral eosinophilia. Based on clinical, histopathological, and laboratory findings, the patient was diagnosed with KD. An itchy subcutaneous mass on the left arm developed at the age of 14 years. Treatment with steroids was effective. However, as the steroids were tapered after the patient developed side effects, the masses relapsed within a few months. Treatment with cyclosporine A was then initiated, which led to an improvement of clinical features and serial levels of cytokines.

**Conclusions:**

We report a rare case of KD with a peculiar clinical presentation. The patient responded well to treatment with cyclosporine A.

## Background

Kimura’s disease (KD) is an inflammatory condition that classically manifests as painless subcutaneous nodules on the head and neck and characteristically occurs in young Asian males of any age [1, 2]. However, ocular involvement of KD is uncommon, especially in children [3]. Additionally, only a small number of KD cases including upper limb lesions have been reported. The pathophysiology of KD remains unknown, but allergic reactions, infections (parasites, viruses, fungi), microbial toxins, atopy, and autoimmunity are considered possible risk factors that may cause activation of the cytokine pathway implicated in KD [2, 4, 5]. Histologically, KD is characterized by lymphoid follicles with germinal centers that are distinguished by an eosinophilic infiltrate and varying degrees of fibrosis [4, 5].

In this report, we describe a unique case of KD in a 4-year-old Japanese boy. The case exhibited bilateral orbital involvement and nodular subcutaneous lesions on the left arm. The patient was successfully treated with cyclosporine A (CsA). To the best of our knowledge, this is the youngest case of KD with bilateral lacrimal gland and arm involvement reported in the English literature.

## Case presentation

**Fig. 1:**
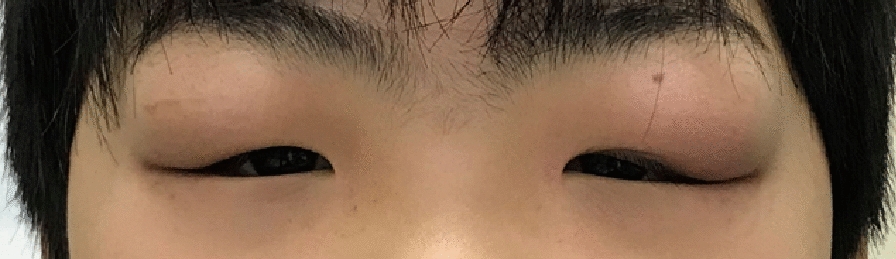
Clinical photograph of the patient. The patient presented with bilateral swollen eyelids

A 4-year-old Japanese boy presented with bilateral upper eyelid swelling and discomfort. The swelling was painless, but he complained of itchy eyelids. Ophthalmic examination revealed bilateral upper eyelid edema (Fig. 1). No lymphadenopathy of the head or neck was observed. The patient had no medical history of atopic dermatitis, but local reactions to insect bites and vaccinations were excessive. There was no family history of autoimmune renal disease.

**Fig. 2:**
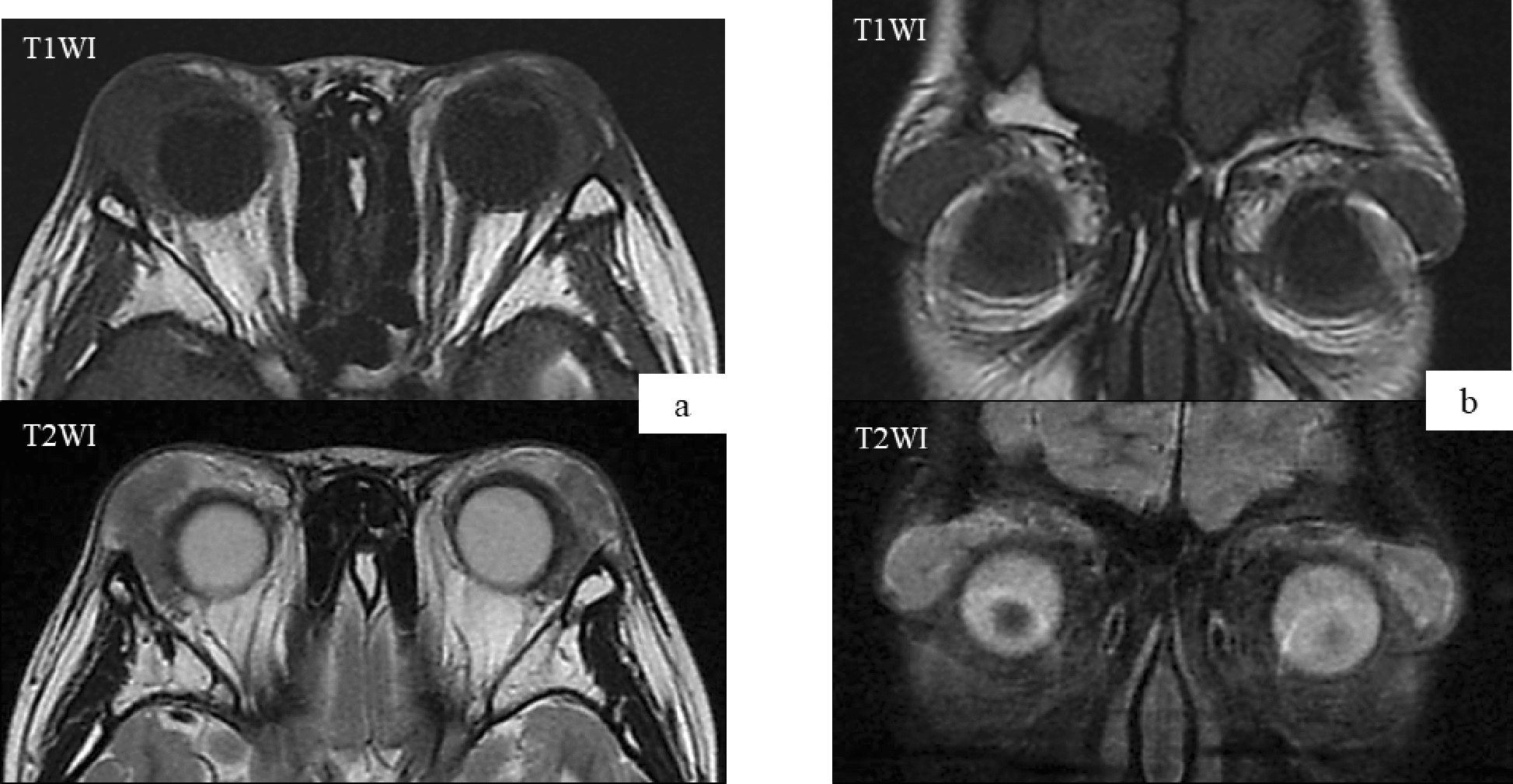
Magnetic resonance imaging. **a**, **b** T2-weighted axial and coronal MRI demonstrated the presence of an enhanced lesion of the bilateral lacrimal gland

Urinalysis results were normal. Blood analysis showed a white blood cell count of 8,200 /mL with a differential neutrophil count of 13%, a lymphocyte count of 55.5%, and a markedly increased eosinophil count of 28.5%. A peripheral smear revealed no abnormalities. The CD4/CD8 ratio was normal (1.46). Most laboratory blood examinations included electrolytes, serum creatinine, and blood urea nitrogen quantitation. The patient had an elevated aspartate aminotransferase level (34 IU/L; normal range, 14–20 IU/L), but alanine aminotransferase activity was within the normal range (16 IU/L; normal range, 10–40 IU/L). Lactate dehydrogenase and creatinine kinase activities were normal. The patient’s C-reactive protein level was 0.045 mg/dL, and he had an erythrocyte sedimentation rate of 9 mm/h. Immunoglobulin levels of IgG, IgA, IgD, IgM, and IgG4 were normal (IgG, 1,173 mg/dL; IgA, 162 mg/dL; IgD, 9.9 mg/dL; IgM 206 mg/dL; and IgG4 9.7 mg/dL). The patient had a highly elevated serum IgE level (14,351 IU/mL; reference range: 0–295 IU/mL). Serologic analyses for antinuclear antibody and MPO-ANCA were negative and serum complement components C3, C4, and total complement activity were normal. Chest radiography results were also normal, though magnetic resonance imaging (MRI) showed bilateral lacrimal gland enlargement without the appearance of mass lesions in the orbit and adjacent bony erosion (Fig. 2).

**Fig. 3:**
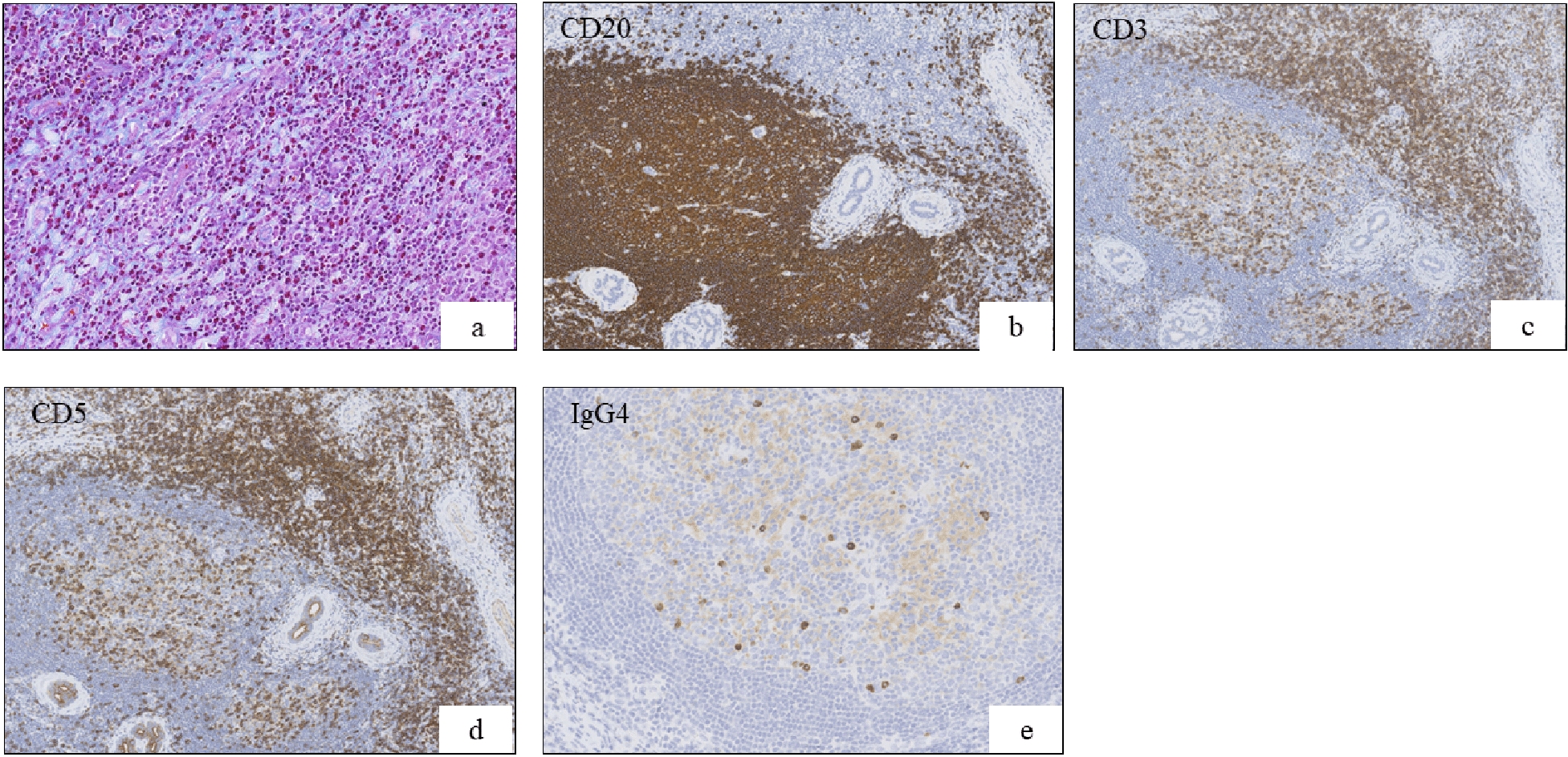
Pathological findings of the lacrimal gland (first biopsy). **a** Diffuse infiltration of the lacrimal gland by eosinophilic hyperplastic lymphogranuloma with fibrosis (Masson’s trichrome staining, 200×). **b**, **c**, **d** CD20-positive B-cells within follicles; CD3-positive and CD5-positive T-cells were present within the interfollicular regions (100×). **e** A small proportion of IgG4-positive plasma cells were observed (200×)

Mikulicz disease was initially suspected, despite the IgG4 level being normal. Based on this presumptive diagnosis, pranlukast hydrate was administered for 2 years. However, follow-up treatment was suspended for 4 years as the symptoms did not improve. An excisional biopsy via anterior orbitotomy was performed at the age of 10 years, which revealed eosinophilic hyperplastic lymphogranuloma involving the lacrimal gland (Fig. 3). A mixture of CD3-positive and CD5-positive T-cells was found within the interfollicular regions, and CD20-positive B-cells were observed within the follicles. A relatively smaller number of IgG-positive and IgG4-positive plasma cells were also observed. There was no evidence of either vasculitis or prominent vascular endothelial cell proliferation.

**Fig. 4:**
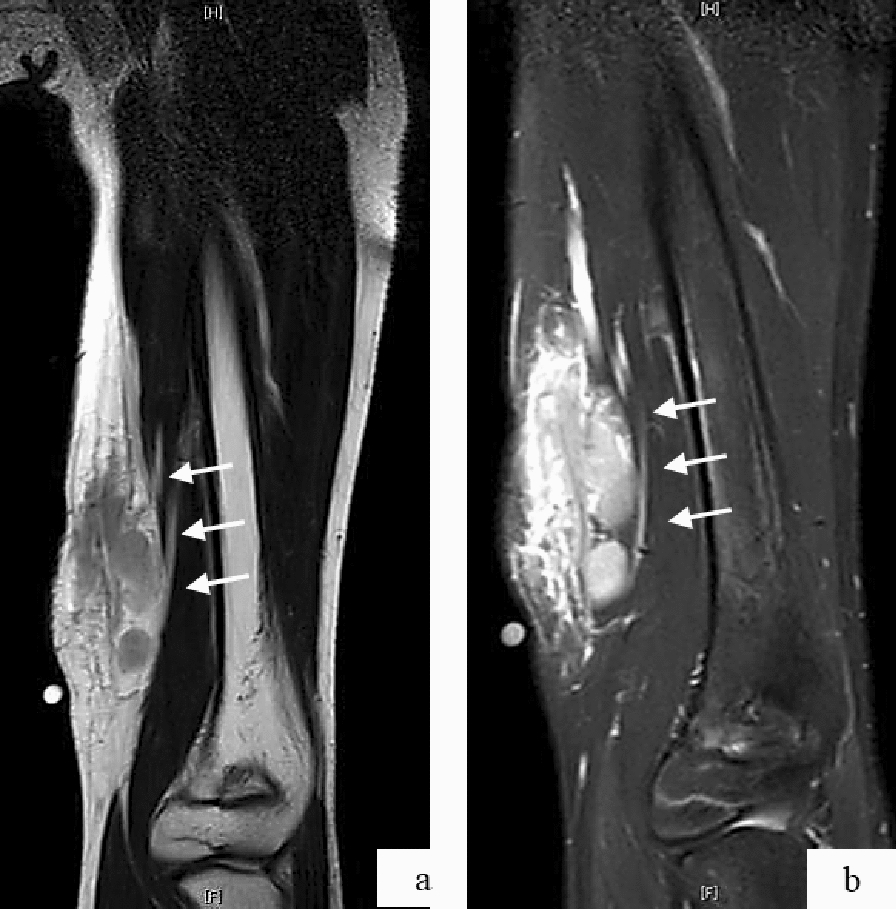
Magnetic resonance imaging of the left proximal arm. **a** T2-weighted coronal MR image reveals the presence of a subcutaneous lesion in the medial aspect of the left distal arm (white arrow). **b** Fat suppression T2-weighted coronal MR image shows the presence with a lesion of complex intermediate signal intensity (white arrow)

**Fig. 5:**
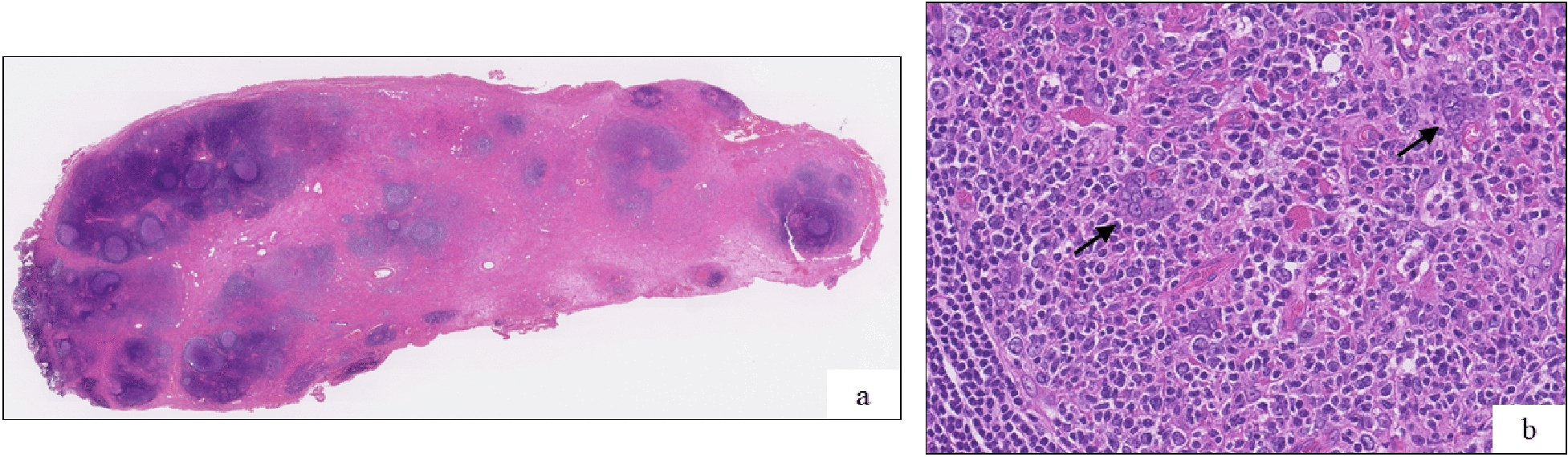
Pathological findings of the lacrimal gland (second operation). **a** Hyperplastic lymphoid follicles embedded in fibroconnective tissue (Hematoxylin and eosin staining, 5×). **b** Warthin-Finkeldey cells are observed within the follicular mantle zones (black arrow) (hematoxylin and eosin staining, 300 ×)

**Fig. 6:**
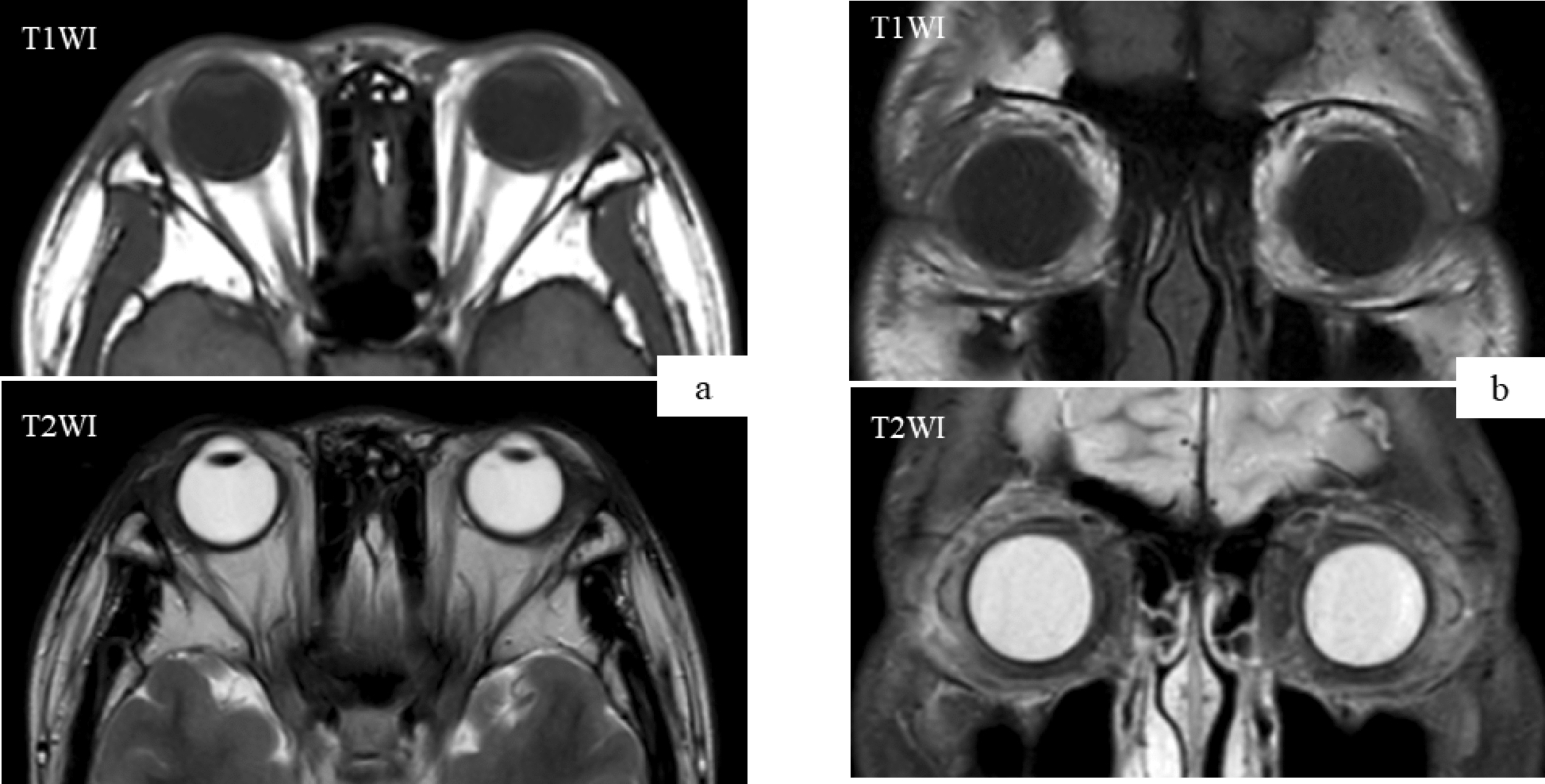
Magnetic resonance imaging (after second operation). **a**, **b** Axial and coronal MRI demonstrated no presence of an enhanced lesion of the bilateral lacrimal gland

Based on these findings, IgG4-related diseases, including Mikulicz disease, were excluded. Oral prednisolone was initiated at 1 mg/kg/day based on a diagnosis of lymphoproliferative disorder. The patient’s symptoms, including the eyelid edema, discomfort, and eosinophilia, were dramatically improved at the 1-month follow-up. However, symptoms recurred as prednisolone was tapered. A combinatorial therapy of prednisolone with mizoribine was subsequently initiated to mitigate the side effects of steroids. However, swelling of the bilateral eyelids worsened, and an itchy subcutaneous mass developed on the left arm. MRI examination revealed serpiginous subcutaneous lesions in the medial aspect of the left distal arm (Fig. 4). The bilateral eyelid masses were resected when the patient was 14 years old due to their frequent recurrence, the side effects associated with steroid administration at the time of recurrence, and the negative impact the masses had on the patient’s physical appearance, and narrow field of view. Postoperative pathological examination of the excited masses showed hyperplastic lymphoid follicles embedded in fibroconnective tissue and multinuclear Whartin-Finkeldey cells, indicative of KD (Fig. 5). Immunohistochemical analysis of the cells revealed negative staining for IgG and IgG4. The serum IL-4 level was elevated (500 pg/mL; reference range: < 6.0 pg/mL), but IL-5, IL-6, and interferon γ levels were normal. Serum sIL-2R levels were with in the reference range throughout the clinical course. The diagnosis of KD was confirmed based on the histological findings, markedly high serum IgE levels, and peripheral blood eosinophilia. Treatment with oral CsA at an initial dose of 2.5 mg/kg/day was started, which maintained at a trough level of approximately 50 ng/ml. The eosinophilia and serum IgE levels gradually decreased after initiation of CsA, but the IL-4 levels remained high. The CsA therapy was adjusted to 4mg/kg/day and the clinical symptoms were stable, without bilateral upper eyelid swelling or recurrence of the subcutaneous mass on the left arm (Fig. 6).

## Discussion and conclusions

Patients with KD almost always have marked eosinophilia and elevated serum IgE levels. Some studies have reported that blood eosinophil counts in patients with KD correlate with lesion size, with larger lesions being associated with higher eosinophil counts [6]. Common symptoms of KD include involvement of the head and neck region, and CT imaging and MRI reveal a wide range of lesions and multiple enlarged lymph nodes [7]. Orbital involvement, including eyelid swelling, is common but has been reported [8–12]. Furthermore, most cases of KD with orbital involvement occur in adults from Asian countries. To the best our knowledge, the case described here is the youngest case of KD with orbital involvement to be reported. Additionally, our patient developed a lesion in his left arm as a result of his condition. The patient opted for the left arm lesion not to be surgically excited as it was asymptomatic, other than itchiness. Involvement of the arm for a patients with KD is rare, but not unique. For instance, Lam et al. previously reported a case of KD affecting the anterolateral aspect of the upper arm [13]. In that earlier case, the skin lesion specimen did not show characteristic findings of KD; however, this may be attributed to the fact that only surface tissue was obtained by biopsy. Similarities in clinical features of our current case and that reported by Lam and colleagues, such as the itchy left arm mass and similar MRI findings, suggest involvement of KD in their case.

Our patient exhibited elevated eosinophil and IgE levels as the eyelid swelling worsened. A related disease was initially suspected, which unfortunately delayed a definitive diagnosis and postponed initiation of the appropriate therapeutic intervention. Clinically, the differential diagnosis of KD may include IgG4-related diseases, Hodgkin’s lymphoma, Castleman’s disease, Langerhans cell histiocytosis, angiolymphoid hyperplasia with eosinophilia (ALHE), drug reactions, and allergies. These histological features may overlap with those of idiopathic orbital inflammation [8, 12] or ALHE [1, 2]. Notably, some cases have been reported of KD occurring in the left lacrimal gland with increased serum levels of IgG4, mimicking IgG4-related disease [14, 15]. In the current case, we made an initial differential diagnosis of IgG4-related disease, despite normal IgG4 levels and lack of IgG4 expression in the lacrimal gland specimen. In the literature, the most common misdiagnosis of KD is ALHE, although patients with ALHE have normal IgE levels and no renal complications [4, 10, 16, 17]. In our case, there were no signs of renal disorders. Histologically, Warthin-Finkeldey cells were not observed in the first biopsy, but were detected in the lacrimal gland tissue following the second operation. Warthin-Finkeldey cells are a giant multinucleate cells found in hyperplastic lymph nodes in the early course of measles, as well as in HIV-infected individuals, neoplastic and non-neoplastic lymph node disorders, and in patient with KD [18–20]. Collectively, the clinical features and other histopathological findings of our case ultimately indicated a definitive diagnosis of KD.

No standard treatment for KD has been established. Suggested therapeutic options include oral or intralesional corticosteroids, surgical removal, and radiotherapy. In most cases, oral corticosteroids are effective as an initial treatment, but relapses have been frequently reported after surgery or withdrawal of oral steroids [21, 22]. Other agents, including CsA, have been reported to induce and/or maintain remission, and there are several reports of treatment with cyclosporine [21–23]. Cyclosporine acts to reduce IL-2 synthesis, resulting in inhibition of T-lymphocyte proliferation and attenuation of the immune response. Sato et al. reported an 11-year-old boy with cervical lymphadenopathy who was successfully treated with steroids and CsA [22]. Similarly, Shin et al. reported an 8-year-old boy with right upper eyelid swelling who was diagnosed with KD and effectively treated with steroids, CsA, and azathioprine [24].

Nephrotic syndrome (NS) has been reported as a complication of KD [21, 25, 26]. Among 11 Japanese patients with KD and NS, two were treated with CsA [4]. Katagiri et al. described how cytokine mRNA levels and eosinophils were suppressed when the blood concentration of CsA was > 75 ng/mL in an adult patient with KD [27]. Marked eosinophilia and elevated serum IgE were associated with regulation of mRNA levels of Th2-cell cytokines, such as IL-4, IL-5, IL-13, and interferon-γ [27, 28]. Furthermore, CsA efficacy may be improved by suppression of these activities in patients with KD [27]. Although sIL-2R levels were normal in the current case, IL-4, peripheral eosinophil counts, and IgE levels were elevated. As in previous reports, administration of CsA improved and maintained the clinical features of the patient, including laboratory findings. Complications of NS during the clinical course of KD with a lacrimal gland mass have been reported previously [25]. Given the high rate of recurrence and reported association with lymphoma [29], along with the possibility of lifelong dry eyes with excision of the mass/lacrimal gland, careful long-term follow-up is recommended for our patient.

In summary, we have described a case of KD with rare or possibly unique clinical manifestations in a patient that would not normally be considered at risk of KD. Indeed, the incongruence with the clinical presentations initially observed in this case led to an inexact diagnosis that delayed initiation of an effective therapeutic intervention for several years. The details of this case provide valuable information regarding the range of clinical presentations that can be associated with KD. Awareness of the details of our case can aid in the timely diagnosis of the patients with KD and increase the efficacy of therapies.

## Data Availability

Not applicable.

## Abbreviations

KD: Kimura’s disease
CsA: Cyclosporine A
MRI: Magnetic resonance imaging
ALHE: Angiolymphoid hyperplasia with eosinophilia
NS: Nephrotic syndrome
